# New Perspectives on the Role of Vitiligo in Immune Responses to Melanoma

**DOI:** 10.18632/oncotarget.323

**Published:** 2011-09-10

**Authors:** Katelyn T. Byrne, Mary Jo Turk

**Affiliations:** ^1^Dartmouth Medical School and the Norris Cotton Cancer Center, Lebanon, NH, USA

**Keywords:** melanoma, vitiligo, melanocytes, CD8 T cells, CD4 T cells, T cell memory

## Abstract

Melanoma-associated vitiligo is the best-studied example of the linkage between tumor immunity and autoimmunity. Although vitiligo is an independent positive prognostic factor for melanoma patients, the autoimmune destruction of melanocytes was long thought to be merely a side effect of robust anti-tumor immunity. However, new data reveal a key role for vitiligo in supporting T cell responses to melanoma. This research perspective reviews the history of melanoma-associated vitiligo in patients, the experimental studies that form the basis for understanding this relationship, and the unique characteristics of melanoma-specific CD8 T cells found in hosts with vitiligo. We also discuss the implications of our recent findings for the interpretation of patient responses, and the design of next-generation cancer immunotherapies.

## INTRODUCTION

The past several decades of research in tumor immunology have revealed a strong link between tumor immunity and autoimmunity. This connection is first understood in the context of the extensive overlap between antigens expressed by a tumor and its normal tissue counterpart [1, 2]. The best-studied example of concurrent tumor immunity and autoimmunity is the development of vitiligo in association with melanoma. Vitiligo, or the autoimmune destruction of melanocytes, is an independent positive prognostic factor for melanoma patients [3-5], and its incidence is increased by certain immunotherapies that drive T cell responses to melanoma [6-8]. For years, autoimmunity has been viewed as a side effect of robust anti-tumor immunity. New research now demonstrates that autoimmune melanocyte killing also directly maintains T cell immunity to melanoma [9]. The crucial role of autoimmunity in shaping anti-tumor immunity now informs the interpretation of immunotherapeutic responses in the lab and in the clinic. In this research perspective, we explore the history linking melanoma and vitiligo in patients, key experiments in animal models that have shaped our understanding of this relationship, and the characteristics of memory T cell responses that are governed by vitiligo. Finally, we discuss the importance of autoimmunity for the success of tumor immunotherapy.

## MELANOMA AND VITILIGO: A HISTORY OF CLINICAL CORRELATIONS

Melanoma and vitiligo, despite diametrically opposed clinical manifestations, have been empirically linked for over 50 years with significant implications for patients. Melanoma, the outgrowth of transformed melanocytes, has long been recognized as a particularly aggressive cancer, with median survival for metastatic disease ranging between 6-11 months [10, 11]. Surgical treatment has been the standard of care for nearly 150 years [12]. One of the earliest case-based observations linking vitiligo and melanoma was published in 1953, when a patient with a ‘melanosarcoma’ developed depigmented lesions [13], followed by a report in 1960 noting systemic depigmentation in a melanoma patient treated with radiation therapy [14]. Four years later, Burdick and Hawk reported regression of cutaneous and visceral lesions – concurrent with the development of vitiligo – in a metastatic melanoma patient treated with Vaccinia virus [15]. The following year, Smith and Sehlin reported the development of vitiligo in several melanoma patients with spontaneously regressing primary tumors [16]. These papers were among the first to suggest that tumor immunity and autoimmunity were linked.

Beginning in the early 1970′s, studies began to suggest that vitiligo portended improved prognosis for melanoma patients. A small study of 11 patients reported that survival was slightly prolonged in metastatic melanoma patients with vitiligo, but concluded that data supporting a ‘direct relationship between the two conditions’ was still lacking [17]. Several case studies continued to comment on the association between vitiligo and enhanced survival in melanoma patients, but without statistical support [18-20]. It wasn't until 1983 that Nordlund and colleagues demonstrated significantly enhanced 5-year survival rates in melanoma patients with vitiligo [3]. This was followed by a more extensive study by Bystryn and colleagues in 1987, which reached similar conclusions [4]. However, these studies included a majority of patients with Stage I or II disease, and data from metastatic melanoma patients was lacking. Recently, in a large cohort of metastatic melanoma patients, Quaglino and colleagues reported that vitiligo was an independent positive prognostic factor correlating with significantly enhanced 5-year survival, providing further support for long-reported clinical observations [5]. Thus vitiligo is clearly associated with improved immunity to melanoma in patients, though the mechanisms linking these phenomena remain a subject of active investigation.

## MECHANISMS OF MELANOMA-ASSOCIATED VITILIGO IN HUMANS

As the correlative relationship between vitiligo and melanoma was beginning to be described, the role of the immune response in melanocyte destruction was also under investigation. In 1971, Milton *et al.* argued that spontaneous regressions of primary melanomas implied active anti-tumor immunity, and suggested that associated vitiligo was also a manifestation of this immune response [17]. Supporting this argument, lymphocytic infiltrates were observed in melanomas of several vitiligo-affected patients that underwent spontaneous regression [16, 21]. The infiltration of lymphocytes into both melanoma and vitiligo lesions, as well as spontaneous regression of primary melanoma tumors [16], led to the hypothesis in 1971 that depigmentation was immune-mediated [17]. Over the next decade, several groups postulated the ‘tempting’ idea that melanoma-associated vitiligo was the result of a cross-reactive immune response [3, 22, 23]. However, early studies conceded that other non-immunological mechanisms may be in play, and alternatives to immune mediated pathology were postulated [24]. Theories suggested that toxins from nerves, free radical scavenging, or byproducts from melanin manufacturing itself resulted in the death of melanocytes or the disabling of melanin production [25, 26].

Today, overwhelming genetic and immunologic evidence supports the autoimmune etiology of vitiligo in melanoma patients. Studies by Houghton *et al.* in the early 1980′s revealed that melanoma cells express a family of differentiation antigens that are shared by normal melanocytes [1, 27]. This family of proteins includes tyrosinase and related proteins TRP-1 (gp75) and TRP-2 (dopachrome tautomerase), as well as gp100, and MART-1 (Melan-A); each of which plays a critical role in melanin synthesis [2]. Melanocyte differentiation antigens have since formed the basis for analyzing antigen-specific immune responses to both melanoma and melanocytes [28].

Currently, there are two major proposed mechanisms of vitiligo pathogenesis; one antibody based, and the other T cell based [29]. Autoantibodies recognizing tyrosinase, TRP-1, and TRP-2, have been detected in the sera of melanoma patients and vitiligo patients [30]. The total levels of antibodies directed against pigmented cells have also been shown to correlate with the extent of vitiligo in melanoma-free patients [31]. Additionally, antibodies found in the sera of vitiligo patients have been shown to lyse melanocytes and melanoma cells *in vitro* [32]. However, data supporting antibody-mediated vitiligo in melanoma patients have been limited. Compared with vitiligo patients, melanoma patients with vitiligo have been shown to have similar titers of antibodies to TRP-2 [33], although they have significantly lower titers of antibodies directed against whole melanoma cells and tyrosinase [34, 35].

On the other hand, there is substantial evidence that CD8 T cells mediate melanoma-associated vitiligo. CD8 T cells taken from lesions of vitiligo patients have been shown to kill melanoma cells *ex vivo* [36], and CD8 T cells from both tumors and peripheral blood of melanoma patients have been shown to kill normal melanocytes [37]. In melanoma patients with vitiligo, clonotypically identical T cells were found in both the tumor and surrounding depigmented lesions [38]. It has been shown that the majority of cells infiltrating these lesions are CD8^+^ T cells that recognize both normal melanocytes and melanoma cells [39]. Accordingly, after therapeutically transferring MART-1 specific CD8 T cells to a melanoma patient, transferred cells were found to accumulate in depigmented lesions that developed as a result of therapy [40]. CD4 T cells have also been found to infiltrate vitiligo lesions, although less is known about melanocyte-specific CD4 T cell responses [36, 41]. Two recent studies have identified increased levels of IL-17 in both the serum and tissue of vitiligo patients [42, 43], which may suggest CD4 T cell involvement. Therefore significant clinical evidence supports the theory that melanoma-associated vitiligo is a CD8 T cell mediated phenomenon, although antibodies and/or CD4 T cells may also play roles.

## LESSONS LEARNED FROM ANIMAL MODELS

Concurrent with these efforts to understand the relationship between melanoma and vitiligo in the clinic, a wealth of mechanistic studies has been undertaken in the laboratory. Inbred C57BL/6 mice and the syngeneic, transplantable, B16 mouse melanoma model have greatly facilitated this work. Hara *et al*. were the first to report the outgrowth of white hair in black mice upon treatment with the TA99 monoclonal antibody to TRP-1 [44]. Mice treated with TA99 were also protected against B16 melanoma, showing for the first time that immune responses against shared melanoma/melanocyte antigens could concurrently mediate tumor immunity and autoimmunity [44]. Since this seminal work, studies have turned largely to inducing CD8 T cell responses, and several MHC I-restricted epitopes from gp100 [45], TRP-2 [46], tyrosinase [47], and TRP-1 [48] have since been employed as targets of melanoma vaccines and cellular therapies. T cell receptor transgenic mice with specificity for melanocyte antigens have also provided a valuable tool for understanding mechanisms driving rejection of established melanoma and the induction of vitiligo [49-52]. Below we discuss the experiments in mice that have most contributed to our understanding of tumor immunity and concomitant autoimmunity.

### Primary T cell responses to melanoma and melanocytes

The major focus of melanoma immunotherapy over the past 20 years has been on breaking T cell tolerance to melanocyte differentiation antigens, and vitiligo is a common occurrence with such therapies. The administration of altered melanocyte antigens has been one strategy used to overcome tolerance. DNA vaccines encoding altered forms of melanocyte differentiation antigens, such as xenogenic gp100 [53, 54], epitope enriched TRP-1 [48], and randomly mutated TRP-2 [55], have each proven effective at priming CD8 T cell responses, resulting in protective immunity against melanoma and concomitant vitiligo. Altered self-antigens have also been highly effective when administered in the context of viral vaccine vectors [56], or as heteroclitic peptides in combination with multifactorial adjuvants such as anti-CD40 and TLR agonists [57, 58].

Other studies have shown that altered antigen is not required to break tolerance to melanocyte antigens if sufficient inflammatory and/or costimulatory signals are present. For example, irradiated B16 tumor cells producing GM-CSF (GVAX) combined with a blocking monoclonal antibody to CTLA-4 breaks tolerance to melanocyte differentation antigens, resulting in protective anti-tumor immunity and vitiligo [59]. Treatment with monobenzone, a compound which results in melanocyte killing through haptenization of tyrosinase and induction of melanocyte autophagy [60], also leads to rejection of B16 melanoma tumors and the development of vitiligo when combined with TLR agonists [61]. Lymphopenia-induced homeostatic T cell proliferation in the context of a progressive melanoma, and the absence of CD4^+^CD25^+^ regulatory T cells (T_reg_), is sufficient to break tolerance and induce vitiligo, likely due to the removal of both suppression and homeostatic cytokine sinks [62]. Thus, inflammatory, costimulatory, and/or cytokine stimuli may be sufficient to break tolerance to antigens expressed by melanoma cells or dying melanocytes.

**Figure 1:**
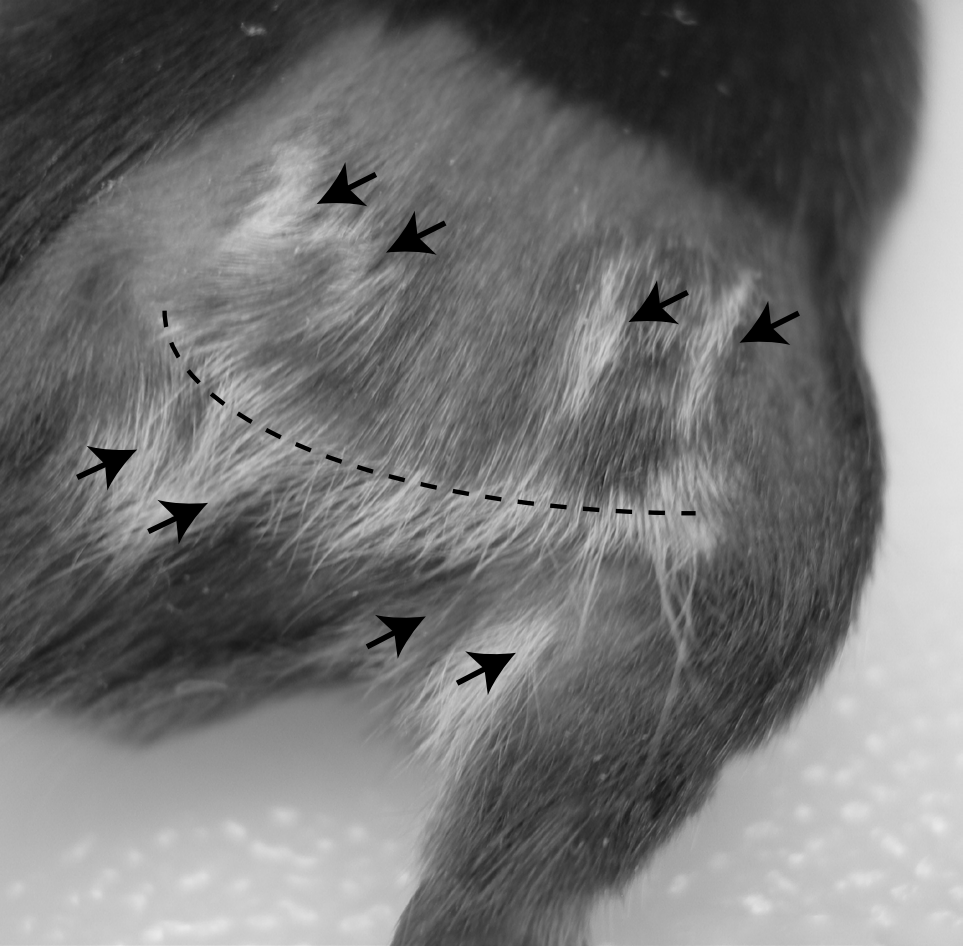
Vitiligo initiation at site of surgery Mice bearing intradermal B16 tumors on the right flank were treated by anti-CD4 monoclonal antibody to deplete regulatory T cells, followed by surgical tumor excision, as we have previously described [63]. 3 weeks after surgery, vitiligo was observed along the surgical incision line (dotted line), and at points where surgical clips had pierced the skin (arrows).

Further, our own work has shown that, even in the absence of overt inflammatory stimuli, disruption of regulatory T cell-mediated suppression is sufficient to overcome tolerance to melanoma-expressed self-antigens. In B16 tumor-bearing mice, we found that depletion of T_reg_ cells by treatment with anti-CD4 breaks CD8 T cell tolerance to TRP-2 and gp100 [63]. These tumor-primed CD8 T cells, although unable to control growth of established primary B16 melanoma tumors, provide systemic concomitant immunity against B16 re-challenge [63]. Furthermore, following T_reg_ depletion, we showed that surgical excision of intradermal B16 melanoma tumors resulted in a majority of mice developing CD8 T cell-mediated vitiligo [9, 63]. Interestingly, trauma to the skin has previously been shown to provide non-specific inflammation that is critical for vitiligo induction [56, 64]. Whereas surgical tumor excision was employed in our model as a means for prolonging host survival, it may also play a role in initiating vitiligo. In fact, we typically observe vitiligo first at the site of surgery [9], often in a pattern that mimics the incision site with striking accuracy (Figure 1). Taken together, these studies indicate the existence of multiple pathways for overcoming T cell tolerance to melanoma and inducing autoimmunity against melanocytes.

In recent years, T cell receptor transgenic (TCR Tg) mouse models have provided crucial new insights into mechanisms driving vitiligo and effective melanoma immunotherapy. The pmel-1 CD8 TCR Tg mouse was the first such model, possessing a TCR specific for gp100_25-33_ in the context of D^b^ [49]. The pmel T cell clone was originally raised by vaccination of wild-type (gp100-sufficient) mice with human gp100, and therefore is thought to have low to moderate avidity [49]. Pmel mice begin to develop mild, spontaneous vitiligo at ~10 weeks of age (KTB and MJT, unpublished observations). Moreover, adoptive transfer of *in vitro* activated pmel cells in conjunction with human gp100 viral vaccination, lymphodepletion, high dose IL-2, and/or TLR stimulation induces profound vitiligo and regression of established melanoma in recipient mice [49, 65-67].

In addition to pmel mice, a TRP-2 specific TCR Tg mouse with specificity for TRP-2_180-188_ (termed Clone 37) was more recently generated [50]. These mice do not develop spontaneous vitiligo, and adoptive transfer of naïve Tg T cells fails to induce rejection of established B16 tumors [50]. The low potency of these cells may be a reflection of a lower avidity TCR, as Clone 37 was originally raised by vaccination of wild-type mice with murine TRP-2 [50]. A third model with specificity for Tyr_369-377_ in the context of HLA-A2.1 expresses the TCR from a CD8 T cell clone that was isolated following vaccination of albino (tyrosinase-deficient) HLA-A2.1 transgenic mice [68, 69]. In contrast to pmel and Clone 37 mice, Tyr_369-377_ TCR Tg mice develop robust vitiligo, with symptoms and kinetics similar to human disease, suggesting that they possess a higher avidity TCR [68].

Interestingly, high-avidity CD4 T cells are also capable of mediating vitiligo, which may relate to the fact that both melanocytes and melanoma cells can express MHC II molecules [70, 71]. A CD4 TCR Tg model with specificity for TRP-1_113-127_ was developed from immunized TRP-1^−/−^ mice [52]. In a wild-type (TRP-1 sufficient) background, TCR transgene expression results in T cell activation and vitiligo development [72], however, these T cells remain naïve on a TRP-1^−/−^ background [52]. Adoptive transfers of low numbers of naïve TRP-1-specific T cells into wild-type mice results in overt vitiligo and potent rejection of established B16 melanoma when given in combination with vaccine and IL-2 [52], irradiation and anti-CTLA-4 [73], or in lymphopenic hosts [72]. Taken together, these TCR Tg models suggest that the development of spontaneous vitiligo in unmanipulated hosts may be dependent on TCR avidity for self-peptide/MHC. These models have proven to be valuable tools for understanding the mechanisms behind effective melanoma adoptive T cell therapy.

### Maintenance of memory T cell responses to melanoma and melanocytes

Although generating robust primary CD8 T cell responses to melanoma has been a subject of intense investigation, less emphasis has been placed on understanding the maintenance of T cell memory, particularly in the context of autoimmune disease. Studies have demonstrated a correlation between long-lived protection against melanoma and depigmentation in experimental models [49, 63, 65, 67], although vitiligo-affected and unaffected mice had not been directly compared with regards to the quality of CD8 T cell memory. Furthermore, whereas the most effective immunotherapies against established B16 tumors clearly generate vitiligo [49, 73], it has remained uncertain whether autoimmunity confers long-term benefits for anti-tumor immunity. Research in our laboratory demonstrated that mice treated by regulatory T cell depletion developed post-surgical CD8 T cell memory responses to TRP-2 and gp100 [63]. Because approximately half of these mice developed vitiligo, this model has enabled investigation of the unique features of T cell memory in hosts with concomitant autoimmunity.

Our recent studies reveal that CD8 memory T cells in vitiligo-affected hosts are potent anti-tumor effectors with key defining characteristics. First, vitiligo-affected hosts maintained gp100 and TRP-2-specific memory CD8 T cells at 10-fold larger frequencies, as compared with unaffected hosts [9]. Second, gp100-specific T cells in vitiligo-affected mice developed an overwhelmingly effector memory (T_EM_) phenotype, in contrast to a predominantly central memory (T_CM_) phenotype in hosts without vitiligo [9]. In accordance with their T_EM_ phenotype, gp100-specific T cells in mice with vitiligo homed preferentially to peripheral tissue sites. Although chronically activated, these cells did not display phenotypic or functional signs of exhaustion, even more than one year after priming [9]. This non-exhausted phenotype was not expected, as it had long been speculated that exposure to self-antigen would drive memory T cells to exhaustion and deletion, which is observed in chronic viral infection models [74]. However, vitiligo-affected mice also exclusively maintained long-term CD8 T cell-mediated protection against a secondary B16 tumor challenge, confirming the protective function of these cells [9]. Studies comparing memory CD8 T cell populations for adoptive T cell therapy had previously led to the hypothesis that T_CM_ cells are more potent anti-tumor effectors than T_EM_ cells [67]. Our studies show that large populations of non-exhausted T_EM_ cells also provide durable tumor protection— a unique feature of hosts with autoimmunity.

### Lessons learned from melanocyte-deficient mice

**Figure 2:**
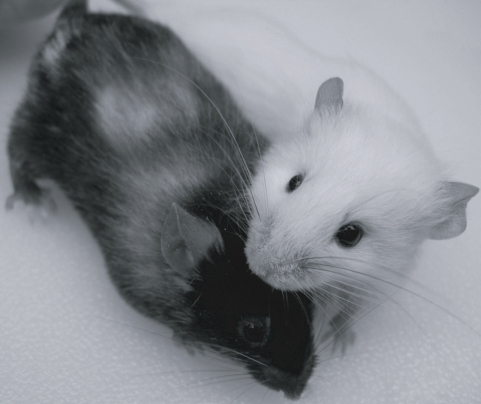
Wsh mice as a model of vitiligo insufficiency A vitiligo affected mouse (*left*) and a Wsh mouse (*right*).

Our comparison of T cell memory in vitiligo-affected and unaffected mice demonstrated a clear correlation between autoimmunity and the maintenance of robust, protective, T cell responses to melanoma. However, these studies did not address whether vitiligo directly contributed to T cell immunity. To specifically answer this question, we established the W^sh^ mouse model of melanocyte deficiency as a model of vitiligo insufficiency (Figure 2) [75]. Due to a mutation in the regulatory region of *c-kit*, W^sh^ mice are overwhelmingly melanocyte deficient, and appear almost completely white, with the exception of the retina [75, 76]. Traditionally used as a mast-cell deficient model, W^sh^ mice had not been previously employed as a model of vitiligo insufficiency [76]. However, these mice have proven to be a useful tool to investigate the role of melanocyte destruction in the maintenance of melanoma T cell responses to melanoma.

Using the W^sh^ model, we have recently found that the absence of melanocytes impairs the development of T cell memory to melanoma [9]. Our data show that W^sh^ mice prime gp100-specific CD8 T cells that are equivalent in frequency and phenotype to wild-type hosts. However, they are unable to develop the robust effector memory T cell responses that characterize hosts with vitiligo [9]. Instead, melanocyte-deficient mice develop small, T_CM_ populations, similar to those in vitiligo-unaffected wild-type mice [9]. Moreover, when gp100-specific CD8 T cells were first primed in tumor-bearing W^sh^ mice, and then adoptively transferred into melanocyte-sufficient mice with vitiligo, the unique T_EM_ phenotype and homing function of these T cells was restored [9]. Thus, the fate of melanoma antigen-specific CD8 T cells was not pre-determined at priming, but rather was altered by exposure to melanocyte destruction. Additionally, robust populations of gp100-specific T_EM_ cells could be rescued in W^sh^ mice by exogenous supplementation of gp100 antigen, confirming that melanocyte antigen is required for the maintenance of T cell memory [9]. These studies establish that melanocyte antigens liberated by vitiligo drive the maintenance of a unique, functional, antigen-dependent memory T cell response to melanoma [9]. The fact that vitiligo directly sustains immune responses to melanoma provides new insight into a phenomenon that has long been observed in the clinic and the laboratory.

### Shared melanoma/melanocyte antigens vs. tumor-specific antigens

While we have emphasized the role of autoimmunity in maintaining anti-tumor immunity, it is important to differentiate between responses directed against shared melanoma/melanocyte antigens and those directed against tumor-specific antigens. In melanoma patients, CD8 T cell epitopes from NY-ESO-1 [2], MAGE [2], and BRAF^V600E^ [77], have been reported, and may represent natural tumor-specific antigens that can be targeted for protective anti-tumor immunity *without* vitiligo. Accordingly, our laboratory has recently shown that protective memory CD8 T cell responses to B16 melanoma can be generated in the complete absence of vitiligo if rejection antigens are tumor-specific [78]. In B16 tumor-bearing mice treated by surgery and a stimulatory mAb against the glucocorticoid-induced TNFR family-related receptor (GITR), we found that protective T cell responses are preferentially directed against tumor-specific antigens [78]. This was evidenced by the fact that mice avidly rejected B16 melanoma re-challenge, but demonstrated no cross-protection against JBRH melanoma. Accordingly, GITR stimulation did not break T cell tolerance to melanocyte differentiation antigens, or induce vitiligo, although it resulted in high-avidity T cell responses against B16 tumor-expressed ovalbumin (OVA) as a model tumor-specific antigen [78]. The mechanisms whereby GITR stimulation skews T cell immunity to tumor-specific antigens remains under investigation, however these data provide evidence that protective CD8 memory T cell responses to melanoma can be generated in the absence of autoimmunity.

There is also evidence that T cell memory to tumor-specific antigens more closely mimics classical memory T cell responses described in acute viral infection models [79]. In studies investigating CD8 T cell responses generated by T_reg_ depletion and surgery in mice bearing B16-OVA tumors, we found that OVA-specific CD8 T cells develop into a small but functional memory T cell population, with a predominantly T_CM_ phenotype [9]. This was true regardless of the vitiligo status of the host [9]. Generating such long-lived T cell memory against tumor-specific antigens will be particularly important for tumors of essential organs, such as the liver or pancreas, where autoimmune responses cannot be tolerated.

**Figure 3:**
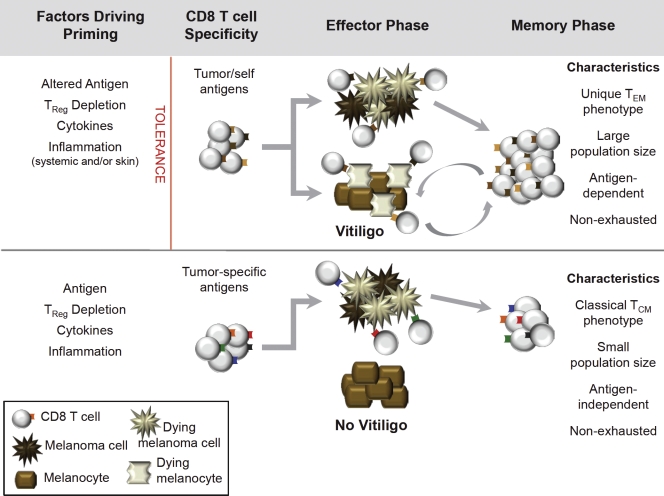
Pathways to protective memory CD8 T cell responses to melanoma (*Top*) Generation of CD8 T cell responses to shared melanoma/melanocyte antigens. Factors that contribute to priming of are listed at left. One or more of these factors leads to the breaking of tolerance (indicated by red line), resulting in effective primary CD8 T cell responses against melanoma that cross-react against healthy melanocytes and induce vitiligo during the effector phase. Ongoing melanocyte destruction provides antigen to support the memory phase of the response; unique characteristics of vitiligo-dependent memory T cells are indicated at right. (*Bottom*) Generation of CD8 T cell responses against tumor-specific antigens expressed by melanoma. Factors contributing to priming are listed at left; there is no need to overcome tolerance to generate primary CD8 T cell responses against tumor-specific antigens. In the effector phase, CD8 T cells directed against tumor-specific antigens kill melanoma cells but do not cross-react with melanocytes, therefore vitiligo does not occur. During the memory phase, CD8 T cells directed against tumor specific antigens possess classical memory T cell characteristics, indicated at right.

For melanoma, we propose that two major types of protective memory T cell responses can be generated, depending on the antigen-specificity of the response (Figure 3). The first type, directed against shared melanoma/melanocyte antigens, has a more stringent set of requirements for T cell priming. However, if T cells efficiently mediate tumor rejection and induce vitiligo, they will develop into a unique, functional memory T cell response that relies on melanocyte destruction for its ongoing maintenance. Alternatively, a second type of memory, directed against tumor-specific antigens, can be likened to T cell responses against foreign antigens. During the effector phase, these T cells will target melanoma cells without killing healthy melanocytes. Subsequently, following melanoma clearance, they develop into classical, antigen-independent T cell memory. As both types of memory would be expected to provide durable protection against melanoma, such a model can explain how long-term immunity to cancer can be achieved either in the presence or absence of autoimmunity.

## SHEDDING NEW LIGHT ON THERAPY-INDUCED VITILIGO IN PATIENTS

Vitiligo has been more difficult to generate in humans than in mice, although certain immunotherapies clearly promote its development. In a large retrospective analysis of 374 metastatic melanoma patients treated with high-dose IL-2, a total of 84 patients (22%) developed treatment-related vitiligo, although in patients with objective clinical responses the incidence of vitiligo was nearly 50% [8]. Another study found that IL-2 and GM-CSF maintenance therapy induced vitiligo in a striking 43% of patients (21 out of 49), who demonstrated significantly enhanced survival as compared with unaffected patients [6]. Combination of high-dose IL-2 with CD8 adoptive T cell therapy (ACT) and lymphodepleting chemotherapy has received much attention in recent years. In a small study, this ACT regimen induced vitiligo in 5 out of 13 melanoma patients, all of whom demonstrated significant tumor regression [7]. More recent data demonstrate that myeloablative regimens further improve the efficacy of ACT, resulting in a 70% metastatic melanoma response rate [80], however, the effects of such therapy on vitiligo development have not yet been reported. These studies demonstrate that high rates of vitiligo can be achieved with certain types of immunotherapy, and may further imply that therapy-induced vitiligo promotes protective immune responses to melanoma. Future investigation of melanoma/melanocyte antigen-specific T cells in surviving vitiligo-affected and unaffected patients should provide further insights into the types of T cell memory that mediate tumor protection in humans.

Vitiligo induction as a crucial component of melanoma tumor immunotherapy is by no means a new concept. In 1977, Lerner and Nordlund suggested that vitiligo should be induced in patients after resection of primary melanoma, and further proposed the use of melanocytotoxic compounds such as monobenzone [19, 81]. Monobenzone therapy was in fact used on a small cohort of late-stage melanoma patients in 1986 and, while 11 out of 17 treated patients had complete responses, their vitiligo status was not reported [82]. No further trials have investigated the therapeutic efficacy of vitiligo induction in melanoma patients, however several groups have resurrected the hypothesis as of late [30, 36, 61, 83]. Recent *in vitro* studies by van den Boorn and colleagues have elucidated the mechanism by which monobenzone results in human melanocyte cell death and the downstream initiation of anti-melanocyte CD8 T cell responses [60]. These studies support animal data from the same group, where monobenzone and TLR agonist treatment of mice with established B16 tumors required CD8 T cells for efficacy [61]. Other methods of specifically targeting melanocytes for destruction, such as anti-TRP-1 mAb [84] and tert-butyl-phenol (TBP) [83] could be used similarly, or as an adjuvant therapy following surgical tumor removal. New mechanistic insights into the importance of vitiligo clearly support future investigation of vitiligo induction as a component of combination melanoma immunotherapies.

Finally, the development of melanocyte-unrelated autoimmune disease in melanoma patients warrants comment. In melanoma patients receiving adjuvant IFN-α therapy, generalized autoimmunity has been reported to correlate with clinical responses [85]. Similarly, following treatment with ipilimumab (anti-CTLA-4), over a third of total responding patients developed autoimmunity, although not limited to vitiligo [11]. While there is no evidence that melanocyte-unrelated autoimmune disease can directly promote melanoma-specific T cell responses, generalized autoimmunity may provide particular inflammatory and/or danger signals that help to overcome tolerance to self-antigens expressed by tumors. Specific attributes of tissue-specific and non-specific autoimmune disease that contribute to anti-tumor immunity are only beginning to be understood, and will require future investigation.

## CONCLUSIONS

The development of vitiligo clearly portends enhanced survival in melanoma patients, and recent experimental data has now provided a new understanding of this phenomenon. Vitiligo is still reported as an ‘adverse event’ during clinical trial evaluation [86], however, this thinking is now shifting. Future studies will be necessary to address whether durable clinical responses in melanoma patients (e.g. those receiving adoptive T cell therapies) are supported by the concurrent progression of vitiligo. More broadly, given the potential efficacy of therapies that target shared tumor/self antigens, we propose that driving tissue-specific autoimmune disease is both feasible and desirable for tumors of non-essential organs (e.g. melanoma, breast, prostate, and ovarian cancer). In conjunction with promising new cancer therapies, harnessing the supportive role of autoimmunity may tip the scales against tumor growth and in favor of lasting anti-tumor immunity.
